# Complications of Diabetes 2017

**DOI:** 10.1155/2018/3086167

**Published:** 2018-03-11

**Authors:** Konstantinos Papatheodorou, Maciej Banach, Eleni Bekiari, Manfredi Rizzo, Michael Edmonds

**Affiliations:** ^1^Outpatient Clinic of Diabetes, University Hospital of Alexandroupolis, Alexandroupolis, Greece; ^2^Department of Hypertension, Chair of Nephrology and Hypertension, Medical University of Lodz, Łódź, Poland; ^3^Diabetes Center and Second Medical Department, Aristotle University of Thessaloniki, Thessaloniki, Greece; ^4^Unit of Diabetes and Cardiovascular Prevention, School of Medicine, University of Palermo, Palermo, Italy; ^5^Diabetic Foot Clinic, King's College Hospital, London, UK

Diabetes is widely recognized as an emerging epidemic that has a cumulative impact on almost every country, age group, and economy across the world. According to the International Diabetes Federation, in 2015, approximately 415 million people were suffering from diabetes worldwide, and this number is expected to exceed 640 million by the year 2040. It is estimated that half of patients with diabetes are unaware of their disease and are thus more prone to developing diabetic complications. However, the cost of dealing with diabetes can be unaffordable in terms of money spent and lives lost. In 2015, approximately 5.0 million deaths were attributed to diabetes, albeit in the same year, more than 12% of the global health expenditure was dedicated to coping with the disease and its complications [1]. Diabetes complications are common among patients with type 1 or type 2 diabetes but, at the same time, are responsible for significant morbidity and mortality. The chronic complications of diabetes are broadly divided into microvascular and macrovascular, with the former having much higher prevalence than the latter [2]. Microvascular complications include neuropathy, nephropathy, and retinopathy, while macrovascular complications consist of cardiovascular disease, stroke, and peripheral artery disease (PAD). Diabetic foot syndrome has been defined as the presence of foot ulcer associated with neuropathy, PAD, and infection, and it is a major cause of lower limb amputation [3]. Finally, there are other complications of diabetes that cannot be included in the two aforementioned categories such as dental disease, reduced resistance to infections, and birth complications among women with gestational diabetes [2]. The present special issue has been devoted to showcase a broad spectrum of research and review papers addressing recent fundamental advances in our understanding of diabetic complications. It includes 12 articles in total, which cover 5 thematic areas: (a) epidemiology and pathogenesis of diabetic complications, (b) microvascular complications, (c) macrovascular complications, (d) miscellaneous complications, and (e) treatment options.

*(a) Epidemiology and Pathogenesis of Diabetic Complications*. The incidence rate of type 1 diabetes varies widely around the world and depends on the interaction between genetic susceptibility and certain environmental factors. Recently, it has been demonstrated that low socioeconomic status is associated with higher morbidity and mortality rates for patients with type 1 diabetes mellitus (T1DM) [4]. In the paper of the present special issue entitled “Incidence and Mortality Rates and Clinical Characteristics of Type 1 Diabetes among Children and Young Adults in Cochabamba, Bolivia,” E. Duarte Gómez et al. tried to determine the incidence, mortality rate, and clinical status of young patients with T1DM in Cochabamba, a province of Bolivia. Supported by a program of the International Diabetes Federation, the authors identified 144 newly diagnosed patients with T1DM and followed them up for over 12 years. The crude mortality rate calculated for this population of T1DM patients was 2.3 per 1000 patient-years. Definitely, this work adds to the very little and relatively old data that was available regarding the prevalence, morbidity, and mortality rates of type 1 diabetes in Bolivia.

It is well established that obesity is a major contributory factor to insulin resistance and type 2 diabetes mellitus (T2DM) [5]. One of the articles of this special issue, by S. Anoop et al., entitled “High Plasma Glucagon Levels Correlate with Waist-to-Hip Ratio, Suprailiac Skinfold Thickness, and Deep Subcutaneous Abdominal and Intraperitoneal Adipose Tissue Depots in Nonobese Asian Indian Males with Type 2 Diabetes in North India,” describes a study designed to examine whether an association exists between plasma glucagon levels and indexes of obesity in patients with T2DM and nondiabetic controls from North India. The authors used anthropometric indexes (body mass index, waist circumference, and truncal skinfolds), dual energy X-ray absorptiometry (DEXA), and MRI scan to determine the status of obesity. They found that nonobese T2DM patients had higher levels of plasma glucagon compared to nondiabetic controls and these levels were positively correlated with indexes of abdominal obesity, subcutaneous abdominal fat, and intra-abdominal fat. With these findings, the authors shed light on the mechanisms by which nonobese individuals may develop type 2 diabetes.

*(b) Microvascular Complications*. Indices of subclinical inflammation, such as higher hsCRP, are correlated with the prevalence of type 2 diabetes and metabolic syndrome [6]. Meprins are metalloproteinases—expressed in kidney by proximal tubules—that have been proven to play a significant role in the development of diabetic nephropathy [7]. In the paper titled “Meprin Metalloprotease Deficiency Associated with Higher Mortality Rates and More Severe Diabetic Kidney Injury in Mice with STZ-Induced Type 1 Diabetes,” J. E. Bylander et al. designed experiments to evaluate the consequences of meprin deficiency in mice with STZ-induced diabetes. Using meprin *αβ* knockout (KO) mice, the authors examined the role of meprins in the development of diabetic nephropathy (DN). They found that mice with the normal meprin gene and severe diabetes exhibited decreased expression of meprin in their kidneys. On the other hand, diabetic meprin *αβ* KO mice had higher mortality rates and greater loss of kidney function compared to those with the normal meprin gene. As the authors suggest, meprins can play a protective role against the development of DN, and this phenomenon may not be restricted to mice. Obviously, these findings are very important and may have useful clinical implications.

Monofilament and vibration perception tests are commonly used screening tools, recommended by several clinical guidelines to detect diabetic peripheral neuropathy (DPN) or even to predict the risk of foot ulcer formation [8]. As the article of this special issue “Diagnostic Accuracy of Monofilament Tests for Detecting Diabetic Peripheral Neuropathy: A Systematic Review and Meta-Analysis” describes, a comprehensive review and meta-analysis were conducted, by F. Wang et al., aiming to evaluate the diagnostic accuracy of monofilament tests in the detection of DPN. Surprisingly, it was found that monofilament testing, used as a screening tool for the detection of DPN, had limited sensitivity, albeit acceptable specificity. The authors support that the use of monofilament testing alone cannot be considered as an optimal practice for the diagnosis of DPN. Dealing with the same issue, J. J. Brown et al., in another paper of this special issue titled “A Comparison of Screening Tools for the Early Detection of Peripheral Neuropathy in Adults with and without Type 2 Diabetes,” investigated the effectiveness of several tools for the early detection of DPN in patients with prediabetes or type 2 diabetes. These tools included vibration perception tests, monofilament testing, the Norfolk Quality of Life-Diabetic Neuropathy (QOL-DN) questionnaire, and the measurement of sural nerve amplitude potential (SNAP) and sural nerve conduction velocity (SNCV) with the help of the NC-stat DPNCheck device. Interestingly, the authors demonstrated a significant positive correlation between 1 g monofilament scores and SNAP values and negative correlations regarding the QOL-DN score and SNAP or SNCV values. These findings provide evidence that the use of 1 g monofilament testing in combination with the QOL-DN questionnaire may be an effective and inexpensive tool for the early detection of DPN in adult patients with prediabetes or type 2 diabetes.

*(c) Macrovascular complications*. Peripheral artery disease is a common complication and comorbidity of diabetes. Patients with diabetic foot ulcers have coexisting PAD at a proportion of approximately 50% and may suffer from chronic ischemic pain [9]. For these patients, pain reduction can improve significantly their quality of life. In one of the papers of this issue entitled “Tapentadol Prolonged Released (PR) Reduces the Severe Chronic Ischaemic Pain and Improves the Quality of Life in Patients with Type 2 Diabetes,” A. Tedeschi et al. evaluated the efficacy and tolerability of tapentadol PR, a drug that acts both as an *μ*-opioid receptor agonist and a norepinephrine reuptake inhibitor, in patients with type 2 diabetes and severe chronic ischemic pain. A numerical rating scale (NRS) and questionnaires, such as DN4 and SF-12 Health Survey, were used to evaluate the analgesic efficacy of the drug. The findings of this study support that tapentadol PR is an efficient drug for the treatment of chronic ischemic pain in patients with T2DM. The authors concluded that not only does tapentadol PR decrease significantly the intensity of the pain but it also relieves neuropathic symptoms and improves the quality of patient's life at the same time.

*(d) Miscellaneous Complications*. It has been demonstrated that patients with diabetes, who experience episodes of severe hypoglycemia, have a higher risk of cardiovascular disease [10]. S. Malkani and A. Kotwal conducted a retrospective chart review study using the electronic database of the University of Massachusetts Medical School Diabetes Clinic. They included in their study only insulin-treated patients with type 1 or type 2 diabetes and analyzed patients' self-monitored blood glucose (SMBG) data and HbA1c levels. In the paper entitled “Frequency and Predictors of Self-Reported Hypoglycemia in Insulin-Treated Diabetes,” the authors noted that hypoglycemia was more frequent among patients with type 1 diabetes, albeit approximately 20% of all the patients with type 2 diabetes experienced one or more episodes of severe hypoglycemia. Moreover, this study documented that glycemic variability is positively associated with the frequency of hypoglycemia in both patients with type 1 and type 2 diabetes. Therefore, targeting glycemic variability may be a reasonable strategy to avoid hypoglycemia in insulin-treated patients with diabetes.

There is cumulative evidence that the formation of advanced glycation end products (AGEs) may play an important role in the impaired wound healing observed in patients with diabetes [11]. Turning their attention to the process of wound healing, Q. Wang et al. in their paper titled “Blocking AGE-RAGE Signaling Improved Functional Disorders of Macrophages in Diabetic Wound” explored the role of RAGE-expressing macrophages in the failure of wound healing in diabetic mice. The authors tried to block AGE-RAGE signaling in the area of the wound by applying topically anti-RAGE antibodies. They noticed that the group of mice that received the treatment with anti-RAGE antibodies showed an acceleration in wound healing. Additionally, immunohistochemical staining revealed an improvement in the phagocytic function of macrophages. Undoubtedly, these results are promising and may have useful clinical implications regarding the treatment of wounds in patients with diabetes.

The goal of the paper entitled “Assessment of Diabetic Cardiomyopathy by Cardiovascular Magnetic Resonance T1 Mapping: Correlation with Left-Ventricular Diastolic Dysfunction and Diabetic Duration” was to evaluate the utility of cardiovascular magnetic resonance T1 mapping as a tool for the diagnosis of diabetic cardiomyopathy (DbCM). With this novel technique, Y. Shang et al. measured myocardial extracellular matrix (ECM) expansion and calculated the derived extracellular volume fraction (ECV) in patients with DbCM and in healthy controls. In addition, they assessed the left-ventricular (LV) diastolic function with transthoracic echocardiographic tissue Doppler imaging. The authors illustrated that subjects with DbCM had significantly increased ECV compared with healthy controls. Moreover, in the DbCM group, the value of ECV was positively correlated with the duration of diabetes and negatively correlated with parameters of LV diastolic function. The present findings demonstrated that measuring the myocardial extracellular matrix expansion with CMR T1 mapping can be a valuable tool for the early diagnosis of diabetic cardiomyopathy.

In another article of this special issue entitled “Thyroid Dysfunction among Greek Patients with Type 1 and Type 2 Diabetes Mellitus as a Disregarded Comorbidity,” M. E. Barmpari et al. investigated the prevalence of thyroid dysfunction in T1DM and T2DM patients from Greece. The authors found that the prevalence of hypothyroidism was not different between patients with T1DM and T2DM, while nodular goiter was more frequent among T2DM patients. Of note, T2DM patients with hypothyroidism had higher HbA1c and total cholesterol levels compared to the euthyroid diabetic patients. Based on these findings, the authors suggest screening for thyroid dysfunction in patients with T2DM because it consists of a frequent comorbidity that can worsen diabetes control.

*(e) Treatment Options*. Hyperglycemia can cause modifications in the eye lens through multiple mechanisms, and thus, patients with type 2 diabetes are at increased risk of developing cataract [12]. L. Ji et al., in their study entitled “Diosgenin, a Novel Aldose Reductase Inhibitor, Attenuates the Galactosemic Cataract in Rats,” investigated the potentially protective role of diosgenin, an aldose reductase inhibitor (ARI), against the development of sugar cataract in rats. The authors conducted two separate experiments. In the first, they administered diosgenin per os to rats and they found that this AR inhibitor delayed the opacification of the lens, which represents an important step in the development of diabetic cataract. In the second, they treated lens epithelial cells (LECs) derived from a cell culture with diosgenin and they observed a decrease in the LECs' osmotic expansion, which is an important process in triggering the onset and progression of diabetic cataract. With their findings, the authors support the use of aldose reductase inhibitors to postpone the occurrence of sugar cataract.

Finally, in the article of this special issue entitled “Dietary Genistein Influences Number of Acetylcholine Receptors in Female Diabetic Jejunum,” S. Schacht et al. examined the effect of genistein, a natural phytoestrogen found in soy, on diabetes-related gastrointestinal dysfunction in mice. Compared with the control group, diabetic mice had fewer acetylcholine receptors (AChRs) in their jejunum and increased distance between consecutive intestinal contractions. Of note, a 4-week genistein-diet reversed the number of jejunum's AChRs, although the effect on the distance between intestinal contractions was insignificant. These results are very encouraging because they illustrate the potential of genistein as a therapeutic agent for the diabetes-related gastrointestinal dysfunction.

*Konstantinos Papatheodorou* *Maciej Banach* *Eleni Bekiari* *Manfredi Rizzo* *Michael Edmonds*
